# Non-pharmacological Treatment Challenges in Early Parkinson's Disease for Axial and Cognitive Symptoms: A Mini Review

**DOI:** 10.3389/fneur.2020.576569

**Published:** 2020-09-25

**Authors:** Gabriella Sharpe, Antonella Macerollo, Margherita Fabbri, Elina Tripoliti

**Affiliations:** ^1^School of Allied Health, Faculty of Health Sciences, Australian Catholic University, Brisbane, QLD, Australia; ^2^Department of Neurology, The Walton Center for Neurology and Neurosurgery, Liverpool, United Kingdom; ^3^Department of Neurosciences, Faculty of Health and Life Sciences, University of Liverpool, Liverpool, United Kingdom; ^4^Clinical Investigation Center CIC 1436, Parkinson Toulouse Expert Center, NS-Park/FCRIN Network, NeuroToul COEN Center, Toulouse University Hospital, INSERM, University of Toulouse 3, Toulouse, France; ^5^Department of Clinical and Movement Neurosciences, Institute of Neurology, University College London, London, United Kingdom

**Keywords:** early Parkinson's disease, speech, swallowing, axial symptoms, cognition, multidisciplinary care

## Abstract

**Background:** Parkinson's disease (PD) is now known to be a multisystemic heterogeneous neurodegenerative disease, including a wide spectrum of both motor and non-motor symptoms. PD patients' management must encompass a multidisciplinary approach to effectively address its complex nature. There are still challenges in terms of treating axial (gait, balance, posture, speech, and swallowing) and cognitive symptoms that typically arise with disease progression becoming poorly responsive to dopaminergic or surgical treatments.

**Objective:** The objectives of the study are to further establish the presentation of axial and cognitive symptoms in early PD [Hoehn and Yahr (H&Y) scale ≤ 2] and to discuss the evidence for non-pharmacological approaches in early PD.

**Results:** Mild and subtle changes in the investigated domains can be present even in early PD. Over the last 15 years, a few randomized clinical trials have been focused on these areas. Due to the low number of studies and the heterogeneity of the results, no definitive recommendations are possible. However, positive results have been obtained, with effective treatments being high-intensity treadmill and cueing for gait disturbances, high-intensity voice treatment, video-assisted swallowing therapy for dysphagia, and warm-up exercises and Wii Fit^TM^ training for cognition.

**Conclusions:** Considering the association of motor, speech, and cognitive function, future trials should focus on multidisciplinary approaches to combined non-pharmacological management. We highlight the need for a more unified approach in managing these “orphan” symptoms, from the very beginning of the disease. The concept “the sooner the better” should be applied to multidisciplinary non-pharmacological management in PD.

## Introduction

Parkinson's disease (PD) is a neurodegenerative disease characterized by a spectrum of both motor and non-motor symptoms. Effective management of patients with PD (PwPD) must encompass a multidisciplinary individualized approach (1–3). Medical management of PD has improved parkinsonian symptoms and quality of life (QoL) either through pharmacological or neurosurgical interventions (4). Nevertheless, there are still challenges in terms of treating the axial (gait, posture, balance, speech, and swallowing) and cognitive symptoms that typically arise with disease progression becoming poorly responsive to both dopaminergic and surgical treatments (5, 6). Particular attention should be paid to axial and cognitive disabilities, which are predictions of dependency development and mortality (7, 8). Identifying the extent to which these symptoms are present in early PD and treating these symptoms early could help reduce their burden in later disease stages. Indeed, the ideal time frame to apply a certain treatment is actually a topic of discussion, especially for trials on disease-modifying treatment (9). What seems to be clear is that if we apply a neuroprotective treatment in early/intermediate PD, it is too late, as the neurodegenerative process is too advanced (9). The same concept could be applied for non-pharmacological treatment for those troublesome symptoms that characterize the advanced PD stage; we need to elaborate and identify strategies that could prevent disabilities and act before their manifested appearance (10).

**Figure 1:**
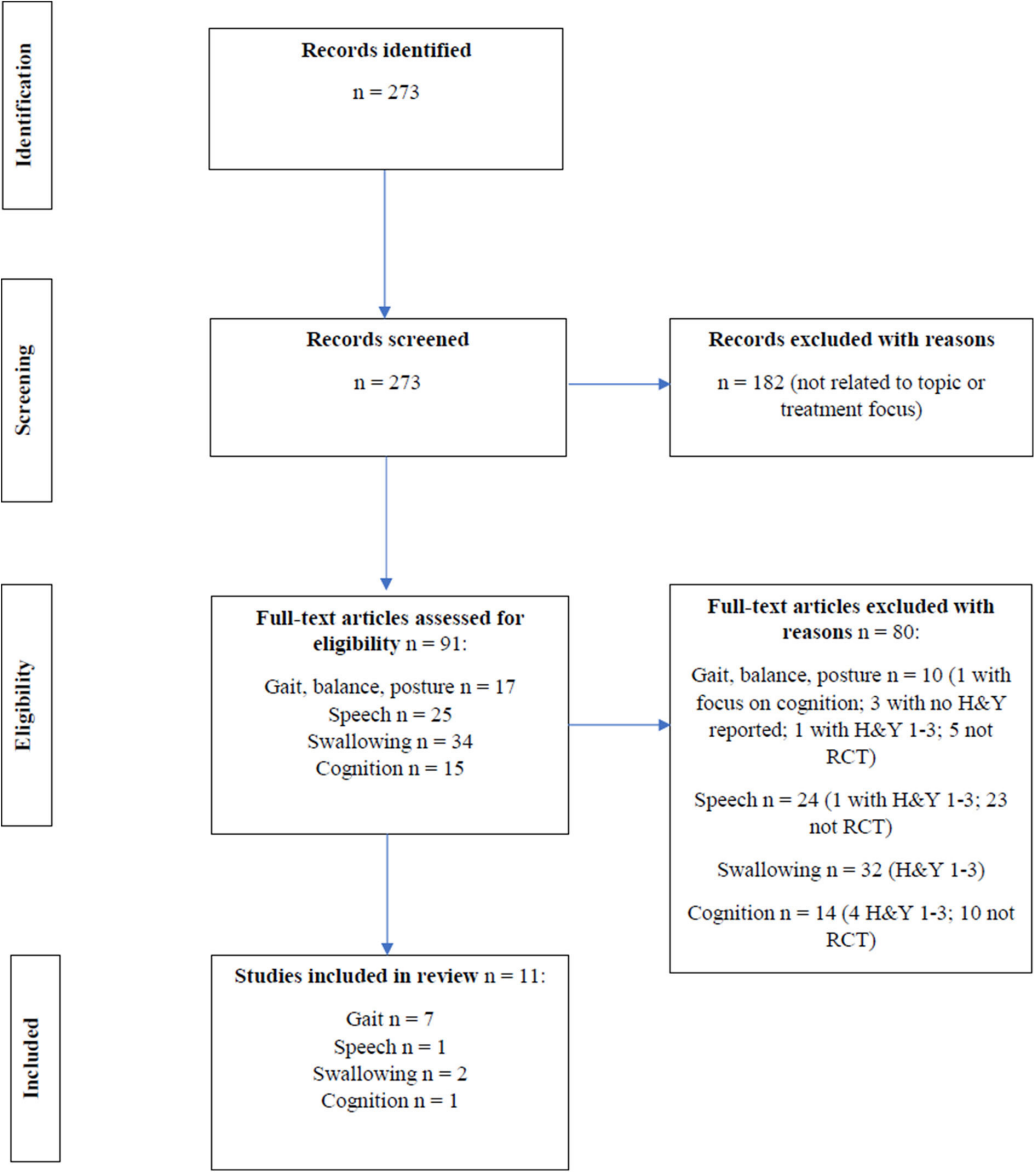
Flow diagram for RCT intervention studies across gait, balance, posture, speech, swallowing and cognition.

In this mini review, the presentation of axial and cognitive symptoms in early PD [i.e., Hoehn and Yahr scale (H&Y) ≤ 2] is reviewed, and the evidence for non-pharmacological treatments for these symptoms in early PD is discussed. Randomized clinical trials (RCTs) on non-pharmacological treatments and written in English from 2005 to 2020 were identified in MEDLINE. Non-invasive brain stimulation was not included in our search. The following search terms (and derivatives) that were used included Parkinson, early, treatment, falls, balance, posture, gait, speech, communication, voice, dysphagia, swallowing, cognition, dementia, and executive function. Relevant treatment studies were selected according to the flow diagram shown in Figure 1. Overall, 11 RCTs (7 studies for gait, one of which had balance as secondary outcome and 0 for posture, 1 for speech, 2 for swallowing, and 1 for cognition) were selected and reviewed (Table 1).

**Table 1:** RCT studies across gait, speech, swallowing and cognition intervention.

**References**	**Participant number; H&Y; disease duration (range or mean)**	**Intervention and follow-up**	**Main outcomes**	**Main results**	**Study limitations**
**Gait intervention**					
Canning et al. (11)	20 (10 per group); H&Y 1–2; duration: 6.1 years	Semisupervised home-based treadmill training vs. usual physical activities 6 weeks, 30–40 min, 4x per week 6 weeks follow-up	6-min timed walk	No significant difference between the two groups (36 m vs. 41.5 m)	Small sample size
Nadeau et al. (12)	93; H&Y 1.5–2; duration not specified	Speed treadmill training (TT, 29 pts) vs. mixed TT (30 pts) vs. controls (light exercise only, 34 pts) 24 weeks, 72 1-h sessions	MDS-UPDRS; PDQ-39; spatiotemporal parameters of gait; 6-min timed walk	No differences between groups on MDS-UPDRS and PDQ-39 (95% CI not stated) Both training groups improved on speed, cadence, and stride length during self-selected walking conditions post-treatment Both training groups improved in distance traveled	Only 34 out of 93 subjects were available for analysis
Agosti et al. (13)	20 (10 per group); H&Y 1.7 ± 0.6; duration: 6 years	Global postural reeducation (GPR) program individual sessions vs. control physiotherapy treatment 12 weeks, 40 min, 3x per week	Kinematic gait parameters of thigh, knee, and ankle	Improvement in kinematic gait pattern with increased flexion amplitudes of knee and thigh post-treatment Significant interaction between time and groups was observed for UPDRS-III score with GPR program	Small sample size; no clear clinically relevant outcome
Pompeu et al. (14)	32; H&Y 1–2; duration not specified	Wii Fit^TM^ games balance training vs. balance exercise therapy (stretching, strengthening, balance exercises) 7 weeks, 1 h, 2x per week	UPDRS-II; Berg balance scale; Static balance using a unipedal stance test	Both groups showed improvement in the UPDRS-II and Berg balance scale No significant difference between groups	No control group without intervention
Fisher et al. (15)	30; H&Y 1–2; duration: <3 years	High-intensity exercise using body weight-supported treadmill training vs. low-intensity exercise vs. zero-intensity education group 8 weeks; 24 sessions; 5 education classes	UPDRS, biomechanic analysis of self-selected, fast walking, and sit-to-stand tasks; corticomotor excitability	All groups showed small improvements in total and motor UPDRS Improvements for high-intensity group only on gait speed, step and stride length, hip and ankle joint excursion during self-selected and fast gait; and weight distribution during sit-to-stand	Small sample size; large variability at baseline
Park et al. (16)	31; H&Y 1–2; duration: <3 years	Early-start group (ESG) or a delayed-start group (DST) exercise program; ESG underwent a 48-week rigorous formal group exercise program for 1 h 3x a week vs. 24–48 weeks of identical program for DSG	UPDRS; get-up-and-go walking test; Tinetti mobility test; PDQ-39, BDI	No significant difference for motor scores between groups, but the ESG group scored significantly better on the BDI	Small sample size; single blinding
Frazzitta et al. (17)	40 (20 per group); H&Y 1–2; *de novo*	Multidisciplinary intensive rehabilitation treatments (MIRT) group vs. control group (only pharmacological treatment) 28-day treatment delivered twice, at 1-year intervals	UPDRS II and III; 6-min timed walk; timed up-and-go test (TUG); PD Disability Scale (PDDS)	Significant improvements over 2 years for MIRT group only on UPDRS II and III, TUG, and PDDS	
**Speech intervention**					
Levy et al. (18)	57 (19 per group); H&Y 2; duration: 4.9–5.0 years	Intensive voice treatment [Lee Silverman Voice Treatment (LSVT LOUD)] vs. intensive articulation treatment (LSVT ARTIC) vs. no treatment controls	Intelligibility measured by transcription accuracy of unfamiliar listeners	Only LSVT LOUD group made significant improvements in transcription accuracy post-treatment	Small sample size
**Swallowing intervention**					
Manor et al. (19)	42 (21 per group); H&Y 2; duration: 7 years	Video-assisted swallowing therapy (VAST) vs. conventional therapy (mainly repeated forceful swallows) 6 sessions each group 1-month follow-up	Degree of reduction of food residue in the larynx using fiberoptic endoscopic evaluation of swallowing (FEES); quality of life—pleasure from eating scale	VAST (education on the swallowing process using visual feedback from patient's own swallowing) was most effective for amount of pharyngeal residue	Small sample size; no long-term follow-up
Baijens et al. (20)	109; H&Y 2; duration: 5 years	Conventional treatment only vs. conventional treatment and surface electrical stimulation (SES)-motor vs. conventional treatment and SES-sensory 15 daily sessions for 30 min; 85 different therapists	FEES and videofluoroscopy (VFS)	Improved swallowing with all treatments No significant differences between treatments	Too many variables, measurements, and therapists; too many muscles in the submental region so high chance for no significance SES tool alone may not be able to trigger changes in the central or peripheral nerve system in Parkinson's disease (PD)
**Cognition intervention**					
dos Santos Mendes et al. (21)	16; H&Y 1–2; duration: 4.7 ± 5.4 years 11 healthy controls	Warm-up exercises and Wii Fit^TM^ training 14 individual sessions 2x week A 60-day follow up	Learning and retention assessed on the scores of 10 Wii Fit games Transfer of learning assessed by the functional reach test	PD participants showed: no deficit in learning or retention on 7 of 10 games; poorer performance on 5 games compared to controls; marked learning deficits on 3 other games associated with cognitive demands Ability to transfer trained motor ability from trained on the games to a similar untrained task	Small participant numbers in each group

*H&Y, Hoehn and Yahr scale; MDS-UPDRS, Movement Disorder Society-Sponsored Revision of the Unified Parkinson's Disease Rating Scale; PDQ-39, Parkinson's Disease Questionnaire (PDQ)-39; BDI, Beck Depression Inventory*.

Gait, balance, posture, speech, and swallowing problems represent the typical axial clinical features of PwPD. Axial impairment is very common among PwPD, inexorably worsening with disease progression, with a severe impact on autonomy and QoL (22, 23). Axial signs are often resistant to pharmacological and surgical treatments. Therefore, non-pharmacological approaches may offer a valuable add-on treatment rescue.

### Gait Symptoms

Gait is controlled by cortical and subcortical neuronal networks, which are both altered in PwPD due to basal ganglia loop disruption and the frontal lobe dysfunction, the latter usually appearing with disease progression (24, 25). However, gait disorders are not only present in advanced PD. It has been shown that in the first 5 years from diagnosis, several gait parameters differ between PwPD and controls, such as lower step width, stride duration, and swing velocity that contribute to reduced gait velocity and a stooped posture (26, 27). Freezing of gait (FoG) can be present in 15–25% of early PD (28, 29). It is associated with the akineto-rigid PD subtype, older age, higher daily levodopa (L-dopa) dose, postural instability, and declining executive function (30), as well as with a higher risk of falls and fear of falling (31). It is often resistant to pharmacological treatment and rarely even worsened by L-dopa (32).

### Gait Intervention

Overall, seven RCTs have specifically looked at gait interventions for early PD, such as treadmill programs, global postural reeducation, high-intensity exercise, or multidisciplinary intensive rehabilitation (11–17). The main findings ranged from no to mild-moderate improvements when compared to traditional care/physiotherapy (Table 1). Studies involving participants at different stages of the disease have also shown similar findings for gait intervention. For example, a Cochrane meta-analysis of 39 RCTs (H&Y 1–4) reported that physical activity and exercise improved gait speed, FoG, and functional mobility over a short term (<3 months) with no clear differences among physiotherapy techniques (33). Further, a recent phase II trial (H&Y 1–3) reported that a high-intensity treadmill program is safe and feasible (34). Finally, cueing has also been shown to be a useful rescue strategy for patients with drug-resistant FoG (35). Recently, performance-based cueing devices tailored to specific patients' gait performance have been developed, thanks to the use of wearable technology (36). The only small cross-over trial study on patients with disabling FoG based in the laboratory setting suggested positive results (37).

### Balance Symptoms

Static balance depends on the interactions between the characteristics of the person, the task, and the environment. Balance disturbances are related to different subsystem dysfunctions, including muscle weakness, proprioceptive sensory loss, increase in sensory thresholds, alteration of motor coordination, and cognitive decline. Interestingly, subtle balance impairments have been observed in patients with idiopathic REM behavior disorder, when compared to healthy controls (38) as well as altered postural sway during cognitive multitasking in early PD (39). For clinical assessment, the most reliable predictive factor of balance impairment is a 12-month history of falls, followed by FoG episodes in the previous month, and comfortable gait speed of <1.1 m/s (40).

### Balance Intervention

Overall, there are no RCTs available on rehabilitation effect specifically for early PD patients. This is partly due to the fact that postural instability is usually clinically relevant in H&Y ≥ 3 patients. Only one single-blind RCT evaluated the effect of Nintendo Wii™-based motor cognitive training vs. balance exercise therapy without feedback or cognitive stimulation on activities of daily living (UPDRS-II). This study also explored the balance Berg scale as a secondary outcome among H&Y 1–2 patients finding an improvement in both groups (14) (Table 1). The most consistent treatment results are seen with motor training and traditional Chinese medical exercise (i.e., Tai Chi and Qigong) as shown by several meta-analyses of randomized RCTs and participants with H&Y 1–4 (41, 42). Of note, reduction in falls has been reported only by Tai Chi trials. It has been recently suggested that aquatic exercise may have equal or greater benefit on balance and fear of falling than land-based exercise (43).

### Posture Symptoms

PD postural abnormalities include: (a) camptocormia, defined as forward trunk bending ≥30° at the lumbar fulcrum (lower) or ≥45° at the thoracic fulcrum (upper); (b) Pisa syndrome, defined as ≥10° of lateral trunk bending that resolve almost completely when lying supine; and (c) anterocollis, ≥45° of forward neck bending. While in advanced stage postural abnormalities may be present in 30% of PwPD, few data is available to characterize postural abnormalities in early PD, which seems to occur in ~5% of H&Y 1–2 patients, and it appears usually isolated (44). Indeed, early and severe postural abnormalities, especially anterocollis, is a red flag for multiple system atrophy diagnosis (45). Multifactorial pathophysiology underlines postural abnormalities, which can be related to a higher disability due to increased risk of falls, back pain, and reduced mobility (46). Postural abnormalities and associated pain treatment remain an unmet clinical need. Camptocormia might improve with dopaminergic treatment, if associated with off periods, or with DBS if it is not yet fixed, while a few positive results have been obtained with botulinum toxin injections for fixed posture.

### Posture Intervention

There are no RCTs available on rehabilitation effect specifically for early PD patients. However, high-intensity programs including stretching, strengthening, gait and balance training, or patient-tailored proprioceptive and tactile stimulation have shown promise in a small group of patients (H&Y 2–4) with moderate postural abnormalities. The positive effect was reported up to 6 months post-intervention (47, 48). However, postural deformities are potentially reversible with early recognition and management. Therefore, patients with mild postural abnormalities might be the best target for rehabilitation.

### Speech Symptoms

Studies have confirmed that speech difficulties are present in early PD, most commonly mild and including features from one or more of the speech subsystems of respiration (e.g., reduced phonation time), phonation (e.g., breathy or rough vocal quality, increased jitter and shimmer), articulation (e.g., imprecise articulation and slow alternating movements), and prosody (e.g., monopitch and monoloudness) (49–56). Variation was noted across studies in relation to the speech subsystem that was most impaired including articulation, phonation, or prosody (52, 55).

Several associations were also found between speech difficulties and/or motor or cognitive abnormalities in early PD. Speech difficulties have been associated with limb bradykinesia and rigidity, more for participants with akineto-rigid motor phenotype (70%) compared to tremor-dominant phenotype (19%) (49). Moreover, monoloudness was found to be correlated to bradykinesia (50), possibly linked to bradykinesia and rigidity at the laryngeal level (53). Furthermore, speech difficulties were found to be a predictor of cognitive decline in PD (53).

### Speech Intervention

Only one RCT is available on the rehabilitation effect specifically for early PD patients. Levy and colleagues (18) found that only the high-intensity voice treatment, Lee Silverman Voice Treatment (LSVT) LOUD®, resulted in significant improvements in intelligibility for patients with mean H&Y 2.1. This was compared to patients receiving a high-intensity articulation treatment or no treatment. The findings are also consistent with the earlier reports from the RCT by Ramig et al. (57) where participants with H&Y 1–3 showed significant improvements in loudness level and functional communication only in the LSVT LOUD® group. Improvements were noted at 1 and 7 months post-treatment.

### Swallowing Symptoms

The most common cause of death in PD patients is aspiration pneumonia, resulting from preexisting dysphagia (58, 59), and therefore, its management should be prioritized. Dysphagia is not just a symptom of late stage PD. In a recent study, Pflug et al. (60) used fiberoptic endoscopic evaluation of swallowing (FEES) on 119 consecutive PwPD and found that 20% of the patients with a disease duration of <2 years had aspiration and that 12% (7 out of 57 patients) with H&Y 2 suffered from severe aspiration. Thus, there is a need to manage dysphagia and avoid complications as early as possible.

A number of existing clinical tools have been shown to be unreliable in detecting PD-related dysphagia. For example, swallowing questionnaires were found to detect swallowing problems in 12–27% of the PwPD, with <10% of the PwPD reporting spontaneously about dysphagia (61–63). Clinical bedside predictors of aspiration used for stroke, like the “normal” water swallow test (64), have been shown to be unreliable in PD (65). The Dutch guidelines estimating the maximum swallowing volume or the maximum swallowing speed (66) were not a suitable screening instrument to predict aspiration in PD patients (60). Increased drooling (sialorrhea) was also deemed a sign of penetration or aspiration (67) until Nienstedt et al. (62, 68) found that drooling cannot be considered an early sign of dysphagia. Therefore, instrumental methods [FEES and Videofluoroscopic Swallow Study (VFSS)] are the most valid and reliable methods of detecting risk of aspiration and penetration in PwPD (67, 68), particularly if they present with the following four symptoms: delayed mastication, reduced lingual motility prior to transfer, aspiration, and total swallow time (69). This information is crucial when evaluating studies on swallowing therapy and the validity of outcome measures.

### Swallowing Intervention

Three RCTs were identified (70), comparing specific rehabilitative techniques to conventional dysphagia therapy, defined as dietary and postural changes. Logemann et al. (71) studied the validity of compensatory strategies and found that the thickness of the bolus is more effective than postural adjustments (“chin down” maneuver) in preventing the incidence of aspiration in the largest sample to date of PwPD (*N* = 711).

Two RCTs have looked specifically at swallowing intervention in early PD. The use of electrical stimulation therapy (SES) by the Baijens et al. (20, 72) was based on the hypothesis that sensory electrical stimulation (delivered through the VitaStim therapy device for 80 Hz, 700 ms, 0–25 mA) on the submental muscles would have a positive effect on FEES and VFS dysphagia ratings. They compared patients (H&Y ≤1–4) undergoing conventional dysphagia therapy with those undergoing sensory- or motor-level stimulation in the submental muscles, 30 min daily for 15 days. There was no significant difference between the groups. This could be due to the lack of physiological rationale over the choice of electrical stimulation in the submental region and the lack of specificity of the muscles stimulated.

Manor et al. (19) compared specific swallowing exercise therapy to conventional therapy and published the results of video-assisted swallowing therapy (VAST) in a group of 21 patients. This therapy was based on the hypothesis that six sessions of visual information and biofeedback from the swallowing process can be more effective than traditional therapy alone. There was a significant improvement in swallowing function from both interventions, with just the pharyngeal residue parameter being significantly better in the experimental group.

The third RCT examined another form of exercise, the strengthening of the expiratory muscles in order to increase the hyolaryngeal movement that protects the airway from liquid or food penetration or aspiration. The expiratory muscle strength training (EMST) device—a calibrated, spring-loaded valve to mechanically overload the expiratory and submental muscles—can improve submental muscle contraction that helps to elevate the hyolaryngeal complex during swallowing and to strengthen the protective cough. In a study of PD participants with mean H&Y of 2.5, Troche et al. (73) compared the EMST with a sham device. The primary outcome measure, the penetration–aspiration score, significantly improved in the EMST group.

### Cognition Symptoms

Mild cognitive impairment with specific involvement of memory, visuospatial, and executive function has been described in early PwPD (74, 75). The executive dysfunction is characterized by deficits in internal control of attention, set shifting, planning, inhibitory control, dual task performance, and on a range of decision making and social cognition tasks (76). Moreover, mild cognitive impairment represents a significant risk factor for early dementia (77). Frontal dysfunction has also been noted using the executive and social cognition battery (78).

Kluger et al. found that fatigue, assessed by the Fatigue Severity Scale, was correlated to reduced visuospatial abilities (79). Cognitive decline in early PD has been associated with worse motor and non-motor symptoms, suggesting that this reflects a faster progressive phenotype (80). Postural control strategies are strictly correlated to cognitive performance, and Fernandes et al. (81) found that cognitive tasks might improve the postural control strategies during gait initiation.

### Cognition Intervention

One RCT has focused on improvements in cognition in early PD based on physical activity. Warm-up exercises and Nintendo Wii Fit™ motor and cognitive skills improved performance in both types of skills in PwPD. However, the ability to learn, retain, and transfer performance improvements after extensive training on Wii Fit games depended largely on the demands, particularly cognitive, of the specific games involved. Thus, those in which patients exhibited no learning deficit have the greatest indication for therapeutic use (21).

Two additional studies with participants at H&Y 1–3 stages have also suggested benefits of physical activity on cognition. Tanaka et al. (82) observed significant improvements in components of executive function on completion of an exercise program. Of note, abstract thinking and the ability to make appropriate decisions under certain circumstances were the primary subsets targeted and improved with the above exercise program. Moreover, the physical activity program resulted in positive trends in verbal fluency and organization of words as well as fewer errors in spatial working memory.

Further, cognitive function showed an improvement after a training period when they were monitored through the Parkinson's Disease Questionnaire (PDQ-39) by Dibble and colleagues (83). Indeed, when PDQ-39 results were compared between PwPD who exercised and those who did not, an improvement in cognitive function was found in the first group.

## Discussion

The findings from this mini review have highlighted that subtle changes in the fundamental areas of axial symptoms and cognition can be present even in early PD (Table 2). Further database searches should be conducted to substantiate the findings from this mini review, which does not aim to present a systematic review of the topic.

**Table 2:** Gait, balance, posture, speech, swallowing, and cognitive symptoms in early PD and current non-pharmacological treatment strategies.

**Symptoms**	**Early features**	**Non-pharmacological approaches/strategies**
Gait	Lower step width, lower stride duration, and slower swing velocity that contribute to lower reduced gait velocity if compared to healthy subjects	High-intensity treadmill, flexibility/balance/function exercise, supervised aerobic exercise
Freezing of gait (FoG)	No specific FoG features in early PD though more responsive to dopaminergic treatment as more related to off periods	Cueing strategies
Posture	Mild stooped posture, generally isolated postural abnormalities (not combined): either isolated PS, isolated camptocomia, or isolated anterocollis	No specific data only for early PD, for H&Y 1–4 PD-positive data for stretching, strengthening, gait and balance training, or patient-tailored proprioceptive and tactile stimulation
Balance	Subtle postural sway	No specific data for early PD; in H&Y 1–4 PD suggested motor exercise (treadmill, walk with auditory/verbal cueing, partnered dance, step training, aerobic exercise, physiotherapy), Tai Chi and Qigong, and aquatic exercise
Speech	Mild difficulties occurring in 1 or more of respiration, phonation, articulation, and prosody Associations between speech and motor or cognition	High-intensity voice treatment (LSVT LOUD) improved intelligibility in H&Y 1–2 and vocal loudness and functional communication for H&Y 1–3
Swallowing	Aspiration detected in 20% of patients within the first 2 years of the disease	Video-assisted swallow therapy (evidence for H&Y2) and expiratory muscle strength training (EMST) (evidence for H&Y 1–3)
Cognition	MCI with involvement in memory, visuospatial, and executive function; MCI as significant risk factor for early dementia Associations between cognitive decline and motor and non-motor symptoms	Improvements in cognition based on physical activity including Wii Fit games and exercise programs

In relation to non-pharmacological interventions over the last 15 years, only a limited number of RCTs have been conducted across areas of axial and cognition with the specific focus on early PD as defined by H&Y ≤ 2. However, in spite of this, positive results have been obtained in the studies, with improvements shown across a range of parameters, allowing to suggest several non-pharmacological approaches for gait, speech, swallowing, and cognitive symptoms in early PD (Table 2). Overall high-intensity treadmill and cueing strategies have obtained some positive results on gait and FoG, Tai Chi and Qigong, and aquatic exercise for balance, LSVT LOUD for speech, VAST and EMST for swallowing and warm-up exercises, and Nintendo Wii Fit™ skills for cognitive symptoms (Table 2). As these symptoms are particularly troublesome and resistant to dopaminergic and surgical treatment in more advanced stages of PD, attention should be given to the development of multidisciplinary non-pharmacological interventions as early as possible before these symptoms become troublesome, as already suggested for palliative care intervention (84). An early treatment approach could help to delay or reduce the high burden and/or medical complications that are often associated with the appearance of these disability milestones in later PD, in spite of the absence of any strong evidences of a clear disease-modifying effect. Although beyond the scope of this mini review, further consideration should also be given to the timing of treatment in early PD and whether the greatest benefit would be for pre-symptomatic or symptomatic patients with PD. In addition, we should clarify if those symptoms need to be targeted directly or rather their risk factors represent the most effective approach. Indeed, we have observed that axial and cognitive symptoms may be subtle in early stage of the disease and eventually difficult to treat or to define a “minimally clinically relevant improvement” for patients. At the same time, recognized risk factors for their occurrence are an older age at disease onset, the need for high L-dopa dose, and the presence of other axial symptoms, such as freezing of gait, falls, or patients who have a postural instability and gait disorder phenotype (30, 85, 86). However, once the abovementioned risk factors are present, it may be too late to achieve optimal gains due to the increased symptoms across axial and cognition domains. This could suggest the need to define if we should apply from the very beginning of the disease specific non-pharmacological strategies to a subgroup of patients with a postural instability and gait disorder phenotype or if we should focus on the pre-symptomatic population, who only present a clinical defined biomarker of possible parkinsonism and dementia development, such as patients with a REM behavior disorder (87).

Early and individualized rehabilitation across the presented axial and cognitive domains would help individuals with PD to maintain a positive impact on QoL and maintain employment and family/social life, while dysphagia and fall management could help to further reduce or delay hospitalization and consequent complications in later stages. A recent longitudinal observational study on the physical activity and early PD has already shown that higher self-reported physical activity is associated with slower disease progression (88). This lends further support to intensive rehabilitation and physical activity in early PD. Despite the few RCTs on early PD, the available evidences suggest that “the sooner the better” is a concept that could be suitable for non-pharmacological multidisciplinary interventions for these four “orphan” symptoms, from the very beginning of the disease.

Further large-scale RCTs are needed to specifically look at interventions for early PD across the areas axial and cognitive domains in a longitudinal manner. To further increase clinical relevance, studies should evaluate treatments that are based on the neurophysiology of PD such as increasing amplitude of movement, sensory–motor calibration, and visual cueing and feedback, using functional outcomes (19, 57, 73). Further, as associations were noted between motor, speech, and cognitive functions, future research should focus on multidisciplinary approaches to combined non-pharmacological management. Advances in this area will continue to empower patients early on in the disease process. The final aim should be to pull together the trends toward a more unified approach in managing these “orphan symptoms” in early PD using a multidisciplinary and individualized care approach that could reduce the disease burden in later PD.

## Author Contributions

ET and GS were responsible for drafting and revising the manuscript, study concept and design, acquisition, analysis and interpretation of data, and study execution. AM and MF were responsible for drafting/revising the manuscript, study concept and design, interpretation of data, and study execution. All authors contributed to the article and approved the submitted version.

## Conflict of Interest

The authors declare that the research was conducted in the absence of any commercial or financial relationships that could be construed as a potential conflict of interest.
